# Targeted Delivery of Human VEGF Gene via Complexes of Magnetic Nanoparticle-Adenoviral Vectors Enhanced Cardiac Regeneration

**DOI:** 10.1371/journal.pone.0039490

**Published:** 2012-07-26

**Authors:** Yue Zhang, Wenzhong Li, Lailiang Ou, Weiwei Wang, Evgenya Delyagina, Cornelia Lux, Heiko Sorg, Kristina Riehemann, Gustav Steinhoff, Nan Ma

**Affiliations:** 1 Reference- and Translation Center for Cardiac Stem Cell Therapy, Department of Cardiac Surgery, University of Rostock, Rostock, Germany; 2 Center for Biomaterial Development and Berlin Brandenburg Center for Regenerative Therapies, Helmholtz-Zentrum Geesthacht, Teltow, Germany; 3 Department for Plastic, Hand, and Reconstructive Surgery, Hannover Medical School, Hannover, Germany; 4 Center for Nanotechnology und Physikalisches Institut, Westfälische Wilhelms-Universität Münster, Münster, Germany; University of Bristol, United Kingdom

## Abstract

This study assessed the concept of whether delivery of magnetic nanobeads (MNBs)/adenoviral vectors (Ad)–encoded hVEGF gene (Ad_hVEGF_) could regenerate ischaemically damaged hearts in a rat acute myocardial infarction model under the control of an external magnetic field. Adenoviral vectors were conjugated to MNBs with the Sulfo-NHS-LC-Biotin linker. *In vitro* transduction efficacy of MNBs/Ad–encoded luciferase gene (Ad_luc_) was compared with Ad_luc_ alone in human umbilical vein endothelial cells (HUVECs) under magnetic field stimulation. *In vivo*, in a rat acute myocardial infarction (AMI) model, MNBs/Ad_hVEGF_ complexes were injected intravenously and an epicardial magnet was employed to attract the circulating MNBs/Ad_hVEGF_ complexes. *In vitro*, compared with Ad_luc_ alone, MNBs/Ad_luc_ complexes had a 50-fold higher transduction efficiency under the magnetic field. *In vivo,* epicardial magnet effectively attracted MNBs/Ad_hVEGF_ complexes and resulted in strong therapeutic gene expression in the ischemic zone of the infarcted heart. When compared to other MI-treated groups, the MI-M^+^/Ad_hVEGF_ group significantly improved left ventricular function (p<0.05) assessed by pressure-volume loops after 4 weeks. Also the MI-M^+^/Ad_hVEGF_ group exhibited higher capillary and arteriole density and lower collagen deposition than other MI-treated groups (p<0.05). Magnetic targeting enhances transduction efficiency and improves heart function. This novel method to improve gene therapy outcomes in AMI treatment offers the potential into clinical applications.

## Introduction

Myocardial infarction is one of the most serious ischemic heart diseases around the world especially in developed countries. Although the effective precaution and medical treatment of myocardial infarction has been improved there are still a considerable number of deaths per year [1].

Surgery intervention as bypass and stenting operation is the first choice for severe coronary heart disease patients. Nevertheless, surgery techniques cannot be applied for all patients. So called no-option patients such as people who already suffer from severe ischemic heart disease may be negatively affected by surgical intervention and eventual death might occur. Therefore, it is necessary to develop a useful strategy for post myocardial infarction therapy of those no-option patients who cannot undergo surgery for revascularization.

Vascular endothelial growth factor (VEGF) plays an important role in repairing the heart after acute myocardial infarction (MI). It has been proved that serum (VEGF) is significantly augmented after myocardial infarction [2]–[4]. A series of research found that after MI, VEGF may interact with VEGFR1(Flt-1) and 2(KDR/Flk-1) which are mostly expressed by endothelial cells and CD34^+^/hematopoietic stem cells and then mobilized and recruited these cells from peripheral blood or bone marrow to ischemic myocardium; After binding to its receptors VEGFR1 and VEGFR2, VEGF could support angiogenesis, inhibit endothelial cell apoptosis and promote endothelial cell proliferation [5]–[7], and restore heart function [8]. Hence, delivery of viral vectors encoding vascular endothelial growth factor gene has showed great potential in the neovascularization of damaged myocardium, ischemia-induced collateral vessel formation and other therapeutic benefits like improvement in cardiac remodeling and left ventricular function [6], [9]–[11].

In general, intramyocardial injection is a useful technique to deliver therapeutic genes to the ischaemically-damaged heart and it has many advantages such as the enhancement of therapeutic gene concentration at target zone. However, the use of intramyocardial injection may be limited in clinical setting because the invasiveness of the surgical intervention might compromise no-option patients. On account of these reasons, intravenous injection as a non-invasive therapeutic method may be a better option to the patients who have advanced heart disease. Intravenous injection has been widely used in MI research in animal model to improve cardiac function [12], [13]. However, the most obviously drawback of the systemic infusion is low targeting therapy. Normally, most systemic infused gene are expressed in liver which plays as a detoxification role [14] and only a very small part of infused vectors is toward to the ischemic heart tissue. A novel technique of magnetic force-enhanced gene delivery in cardiovascular system showed a great potential for rapid and efficient transfection [15]–[17]. Compared with intramyocardial injection therapy, magnetically-based gene delivery is less invasive and safe. Furthermore, magnetic nanoparticles and magnetic field are suitable for biological application, because they are well tolerated and do not interfere with biological processes of the body [18], [19]. Magnetic nanoparticles can be conjugated with therapeutic genes and intravenously injected. After delivery to the peripheral blood, the conjugated genes can be directed to the target site by magnetic force.

In this study, we developed stable magnetic nanobead/Ad complexes and verified its feasibility of delivering therapeutic gene to the infarcted heart by external magnetic guidance for AMI treatment.

## Materials and Methods

### HUVECs isolation and viral vector production

Human umbilical vein endothelial cells (HUVECs) were isolated from umbilical cord, expanded, and characterized by immunohistochemical staining with antibodies against CD31, CD34, CD45 as described earlier in our lab [20]. Briefly, umbilical cords were washed with 0.2-mm-filtered cord buffer (140 mM NaCl, 4 mM KCl, 2 mM MgCl2, 11 mM glucose, 10 mM HEPES [pH 7.4]) and reperfused with 0.2-mm-filtered cord buffer containing 1% bovine serum albumin (SigmaeAldrich, USA) and 0.05% Chlostridium histolyticum collagenase (SigmaeAldrich, USA). The cord was clamped and incubated at 37°C for exactly 13 min. After incubation, the collagenase solution was eluted and HUVECs were purified by plastic adherence. At 37°C and a humidified atmosphere containing 5% CO2 we cultured 2.5×106 cells/cm2 in RPMI 1640 medium (Lonza, USA) containing 20% fetal calf serum (FCS; inactivated at 56°C for 20–30 min; PAN Biotech, Germany), 5% endothelial mitogen (Biomedical Technologies, USA), 100 u/ml Penicillin, 14 mM HEPES (pH 7.4) and 2 mM L-glutamine until cells formed colonies to 90% confluency. Cells were harvested using trypsin and subsequently replated at 5×103 cells/cm2 in endothelial medium (EGM-2; Lonza, USA) for continued passage. Medium was changed three times per week. After HUVECs became confluent to 90%, cells were kept in liquid nitrogen for long-term storage. After third passage, cells have been used for subsequent in vitro experiments.

Ad5.CMV (Vector Biolabs, Pennsylvania, USA) is derived from adenovirus serotype 5 with the deletion of the viral E1 and E3 genes. These adenoviral vectors could carry LacZ, GFP, luciferase and hVEGF gene seperately under the control of the human cytomegalovirus (CMV) immediate-early promoter with a polyadenylation site. These viral vectors were produced by using 293A cells (Invitrogen, USA), a subline of 293 cells (human embryonal kidney cells transformed by sheared adenovirus serotype 5 genome), and purified by adenovirus Purification Kit (Clotech, Japan). The original preparation was at a concentration of 1.0×10^10^ pfu/ml and stored frozen at −80°C.

### MNBs/Ad complexes formation and characterization

Sulfo-NHS-LC-biotin (Pierce Chemical, USA) was used as the biotinylation reagent. Sulfo-NHS-LC biotin were added to 500 μl (1×10^10^ pfu/ml) of an Ad solution in PBS (pH 7.6) to final concentrations (500 ng/ml). The mixtures were placed on ice, in the dark, for 2 h, and then 90 mM glycine in PBS (pH 7.6) was added to each reaction mixture to absorb unreacted sulfo-NHS-LC-biotin. Three rounds of ultrafiltration using ZM-500 centrifugal filtration units (molecular mass cutoff, 500 kDa; Millipore) with PBS (pH 7.4) containing 0.05% Tween 20 (PBST) were used for the removal of non-virion-associated biotinylation reagent. For the next step, appropriate a amount of Streptavidin MagneSphere® Paramagnetic Particles with average effective diameter of 100 nm (Promega, Madsion, WI, USA) were added to biotinylated adenoviral vectors and were provided by vortexing for 30 s and followed by incubation at room temperature for 30 mins. The resulting MNBs/Ad complexes are stable in aqueous solution and can be stored at −80°C for several months. MNBs/Ad complexes size was measured with ZetaPALS analyzer (Brookhaven Instruments Coporation, USA) at 25°C. The complexes solution was diluted with PBS to the final amount of Ad of 8×10^7^ pfu/cm^2^.

### *In vitro* transduction

*In vitro* transduction efficiency of Ad or MNBs/Ad encoded luciferase (Ad_luc_) complexes were evaluated by the luciferase reporter gene in HUVECs. The cells were maintained in endothelial medium EGM-2 (Lonza, USA) supplemented with 100 μg/ml of streptomycin (PAA, Coelbe, Germany) at 100 units/ml of penicillin (PAA, Goelbe, Germany), at 37°C in a humidified 5% CO_2_ incubator. Cells were seeded in 48-well plates at a density of 5×10^4^ cells per well 24 h prior to the transduction. During the transduction, the culture medium was replaced with 0.5 ml complete EGM-2, meanwhile a mount of MNBs/Ad_luc_ complexes were added to the fresh medium. The cells receiving Ad_luc_ alone were taken as control. Cell culture dishes were kept with a magnet placed underneath for 30 min. The sintered neodymium-iron-boron magnets (NeoDelta; remanence Br: 1080–1120 mT) were obtained from IBS Magnet (Berlin, Germany). The sizes of the cylindrical magnets were 6×5 mm (diameter × height). Cells were incubated with complexes for 24 h at 37°C. Following the incubation, the cells were washed with PBS and lysed with 100 μl of cell lysis buffer (Promega, USA). Luciferase activity in cell extracts was measured for 10 s using the luciferase reporter assay kit (Promega, USA) on a 96-well microplate luminometer (MicroLumatPlus LB 96V, EG&G Berthold, Bad Wildbad, Germany) [21]. The relative light units (RLU) were normalized against protein concentration. Protein concentration was measured by a microplate reader using the BCA protein assay kit (Pierce, USA).

### MTT cytotoxicity

HUVECS were seeded into the 96-well plate. The MNBs/Ad_luc_ complexes were added 24 hours after seeding. After 40 hours of incubation at 37°C, 15 μl MTT (5 mg/ml in 1×PBS) was added into each well. And after another 4 hours of incubation at 37°C, the medium was removed and the purple crystals were dissolved in 100 μl dimethylsulfoxide (DMSO). Absorbance was measured at a wavelength of 550 nm and a reference wavelength of 655 nm using a microplate reader (Model 680, Bio-Rad). The results were expressed as the percentage of viability with respect to the control cells, which were cultured without complexes treatment [22], [23]. Cell viability was calculated using the equation below: Cell Viability(%) = (OD_550_−OD_655_) samples/(OD_550_−OD_655_) control×100%.

### Magnetic field guided *in vitro* transduction

HUVECs were incubated for 24 h with MNBs/Ad encoded lacZ (Ad_lacZ_) complexes by the reporter gene LacZ in the presence of three cylindrical magnets (each 6 mm in diameter) affixed to the bottom of a 10 cm culture dish. Three cylindrical magnets were evenly attached to the bottom of the dish to form a three-point magnetic field pattern prior to the application of MNBs/Ad_lacZ_ complexes. The positions of three magnets were adjusted to exclude the possibility of passive accumulation of complexes in the center or along the peripheral edge of the dish. The dish was subjected to gentle agitation at room temperature for 30 min and further cultured for 48 h without the magnets. Then the cells were fixed and stained for light microscopic evaluation of LacZ gene expression.

### MI and intravenous Ad infusion

For this study we used Lewis rats which were purchased from Charles River Laboratories (Sulzfeld, Germany). The federal animal care committee of the LALLF Mecklenburg-Vorpommern (Germany) approved the study protocol (approval number LALLF M-V/TSD/7221.3-1.1-080/11). Lewis rats (male, 250±5 g) were randomly assigned to 7 groups. Rats were anesthetized intraperitoneal with Ketamine (60 mg/kg bodyweight)/Xylazin (10 mg/kg bodyweight). Body temperature was maintained at 37.5 degree±0.5 with heating table. During the first 4 postoperative days, rats were offered NovaMin sulfone (metamizol) (10 drops/300 ml water) for pain-free, and the drinking water was replaced daily. During the entire period of the project, the animals were maintained in accordance with the requirements of the Animal Welfare Act in good faith and with great care. Here we used generally accepted criteria to assess the condition of the animals including exercise, activity, posture, behavior, lethargy, nutrition, lack of appetite, weight, hair texture and body temperature. The rats were euthanized if they showed significant impairments.

**Figure 1:**
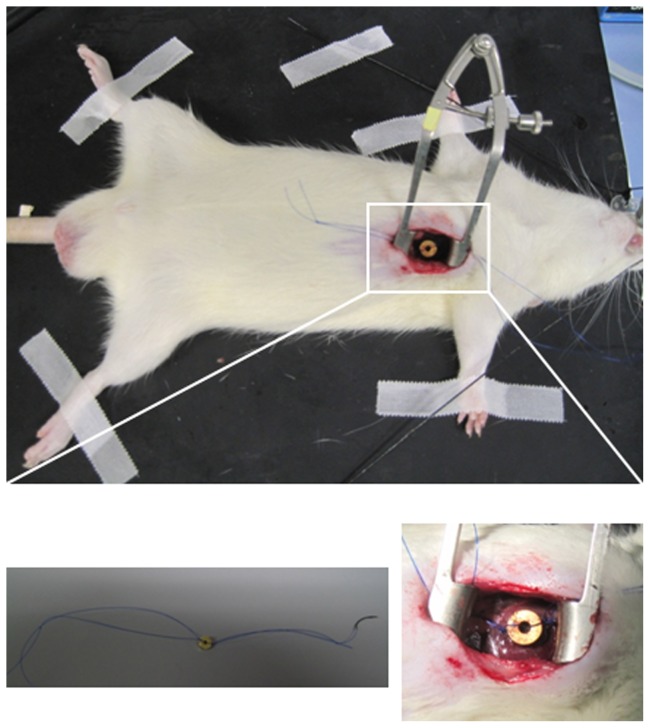
Experimental design. After LAD-ligation rats received the magnet fixed onto the area of blanched myocardium.

Rats were randomly assigned to 7 groups as follows: MI-Magnet^+^MNBs/Ad_hVEGF_ (n = 18);

MI-Magnet^−^MNBs/Ad_hVEGF_ (n = 18); MI-MNBs (n = 18); MI-Saline (n = 18); sham-operated (n = 13); MI-Magnet^+^MNBs/Ad_GFP_ (n = 8); and MI-Magnet^−^MNBs/Ad_GFP_ (n = 8). In MI-Magnet^+^MNBs/Ad_hVEGF_, MI-MNBs, and MI-Magnet^+^MNBs/Ad_GFP_ groups, MI was induced as described earlier [24]–[26]. Briefly the left anterior descending coronary artery(LAD)was ligated permanently. And a 6×2×2 mm cylindrical NdFeB magnet (Br: ≈1000 mTesla) (IBS, Germany) was placed in the chest of the rat closely adjacent to the infarcted area of the heart, then the magnet was fixed between the third and fourth ribs of rat by 4-0 suture (Figure 1). In MI-Magnet^−^MNBs/Ad_hVEGF_ and MI-Magnet^−^MNBs/Ad_GFP_ groups, rat was identical operated and a similar size ceramic bar was placed. And saline group underwent the identical surgery procedure with LAD ligation. Sham operation was performed on another group of rat by passing a suture around the LAD without ligation. 24 h after infarction, Ad encoded hVEGF (1×10^10^ pfu/ml) or GFP (1×10^10^ pfu/ml) coupling to 500 µl MNBs, MNBs alone, or saline were injected in a total volume of 1 ml through the tail vein. 48 h and 7 d after Ad injection, animals from each groups (n = 4) were sacrificed and examined for hVEGF and GFP expression, distribution and inflammatory respond. The remaining animals were used for histopathological analysis and evaluation of heart functioning.

**Figure 2:**
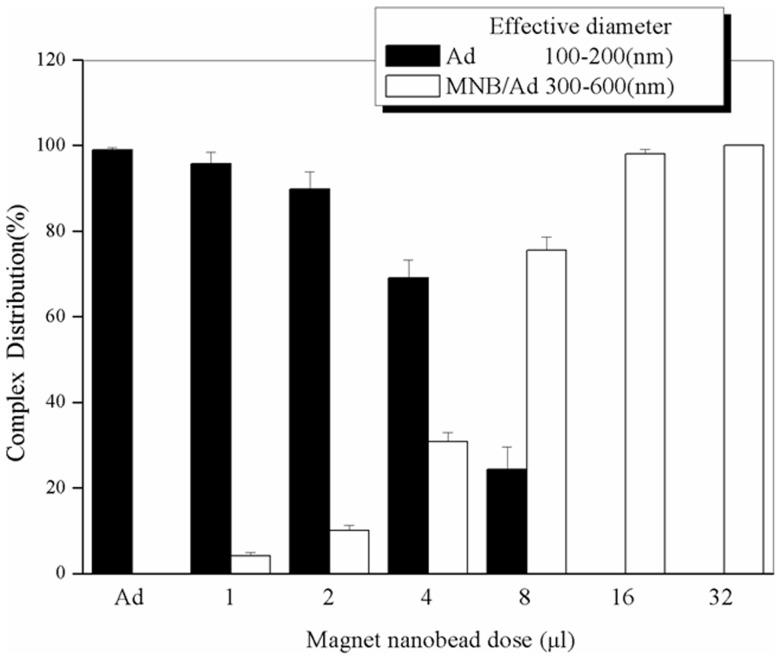
Characterization of MNBs/Ad complexes. Ad concentration (expressed as Ad amount in unit area (pfu/cm^2^)) was 8×10^7^ pfu/cm^2^ and MNBs dose were varied. MNBs/Ad complexes group was increased whereas the naked Ad group was reduced with the increase of the MNBs dose. The complexes group became close to constant 100% from the MNBs dose at 16 µl. Data are expressed as the mean ± SD. (*n* = 6, three independent experiments).

**Figure 3:**
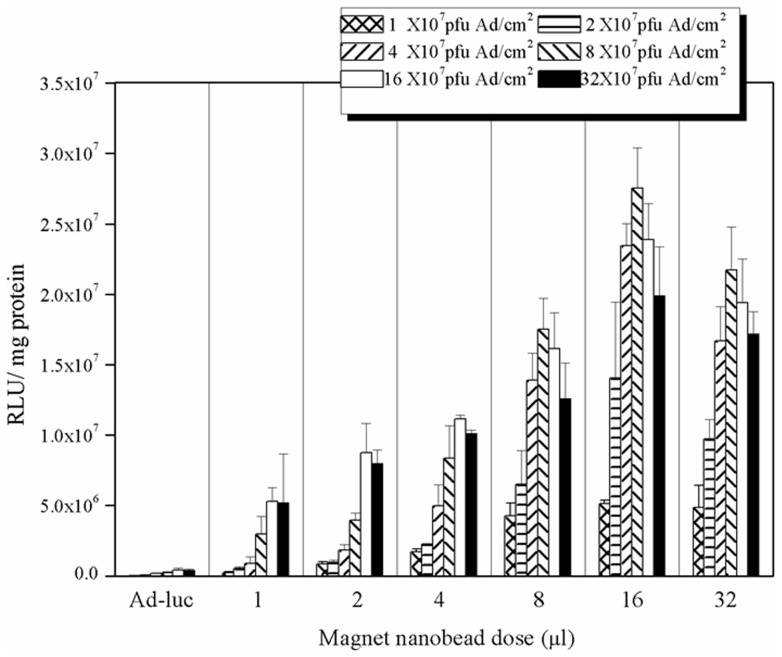
*In vitro* Luciferase expression of HUVECs transduced by MNBs/Ad_luc_ complexes and evaluation of magnetically controlled gene delivery. Tranduction efficiency enhanced by MNBs. Transduction was carried out in 48-well plates. Ad (expressed as Ad amount in unit area (pfu/cm^2^)) and MNBs dose were varied. RLU were normalized to the protein content of cell lysates. Data are expressed as the mean ± SD. (*n* = 6, three independent experiments).

**Figure 4:**
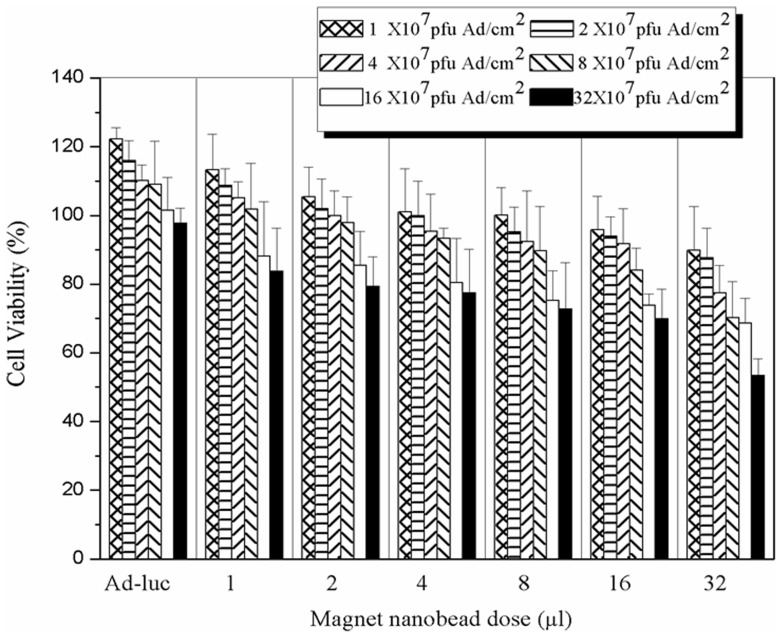
Cytotoxicity of MNBs/Ad_luc_ complexes in HUVECs. Ad (expressed as Ad amount in unit area (pfu/cm^2^)) and MNBs dose were varied. Values were expressed as a percentage of viability of MNBs/Ad_luc_ treated cells relative to that of Ad_luc_ alone treated cells (control). Data are expressed as the mean ± SD. (*n* = 6, three independent experiments).

**Figure 5:**
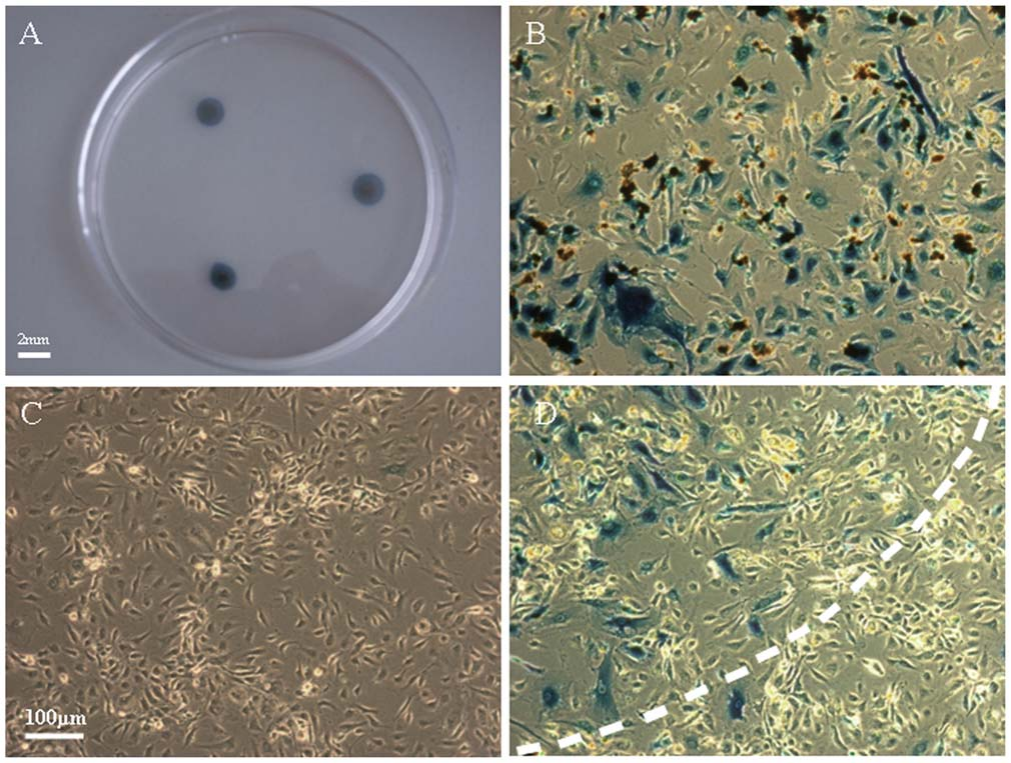
In *vitro* LacZ expression of HUVECs tranduced by MNBs/Ad_Laz_ complexes and evaluation of transduction specificity. (A) The transduced HUVECs formed a three-spot pattern determined by the locations of the magnets. (B–C) LacZ reporter gene expression in the confined area influenced by the magnetic field and in an area remote from the magnetic field. (D) The expression of reporter gene LacZ was detected and formed a clear border between the transduced and untransduced cells.

**Figure 6:**
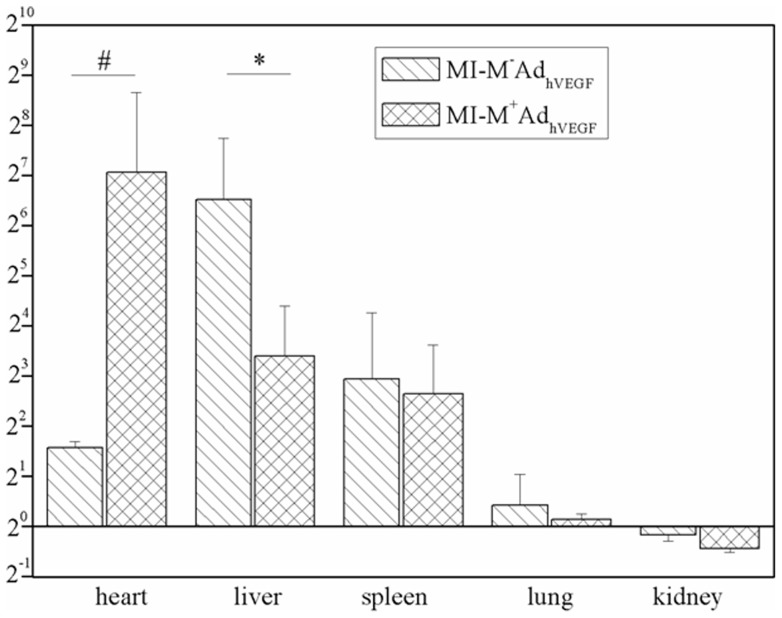
Real time PCR analysis for the distribution and expression of magnetically controlled gene delivery after systemic infusion of MNBs/Ad_hVEGF_ or MNBs/Ad_GFP_ complexes. Quantitative Real-time PCR analysis for hVEGF gene expression in hearts, livers, spleens, lungs and kidneys in MI-M^+^MNBs/Ad_hVEGF_, MI-M^−^MNBs/Ad_hVEGF_ and MI-saline groups. *P<0.05 versus heart in MI-M^−^MNBs/Ad_hVEGF_, #*P*<0.05 verus liver in MI-M^−^MNBs/Ad_hVEGF._ (MI-M^+^MNBs/Ad_hVEGF_, n = 4; MI-M^−^MNBs/Ad_hVEGF_, n = 4; MI-Saline, n = 4).

**Figure 7:**
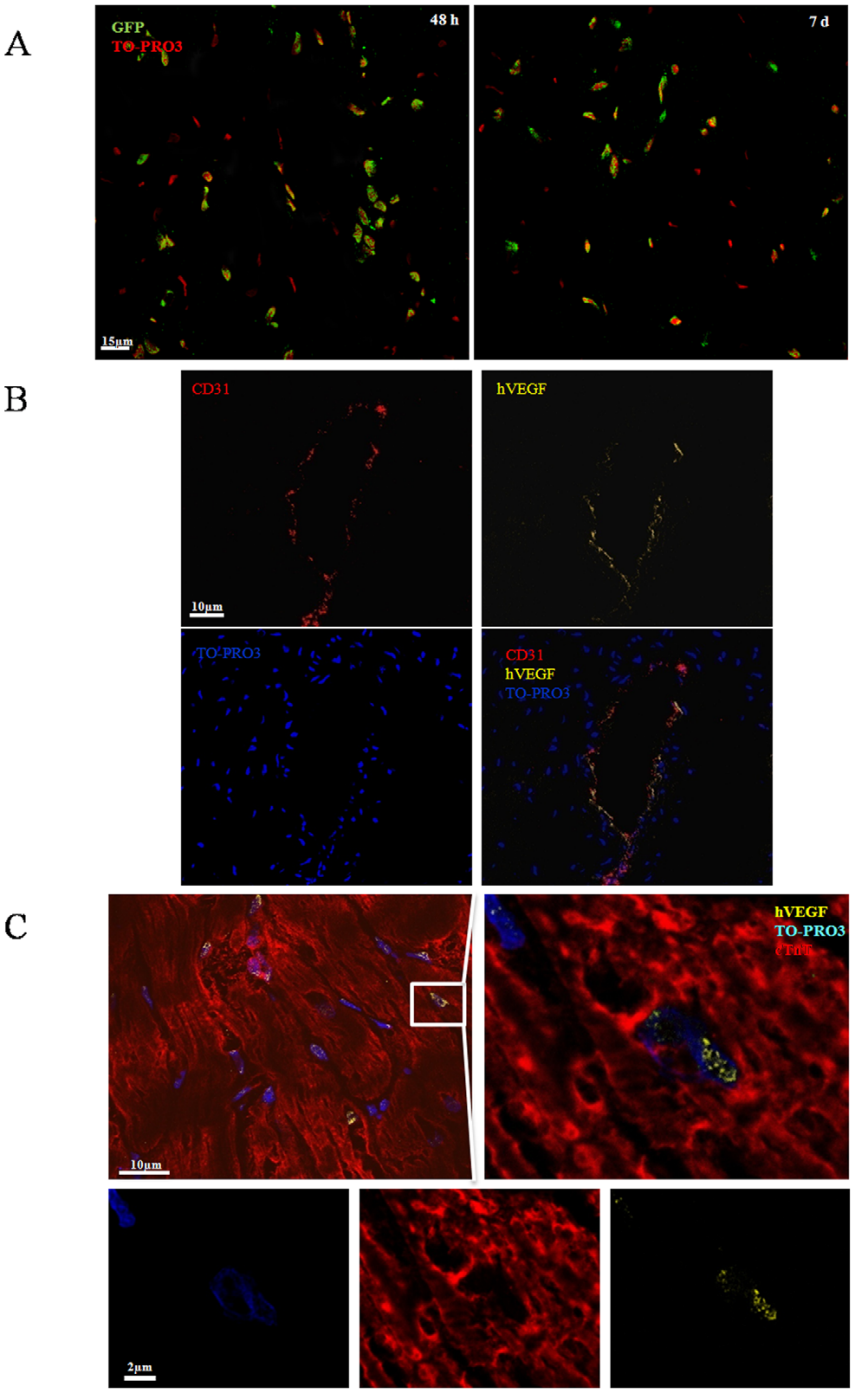
Immunohistologic analysis for GFP and VEGF expression in the infarcted myocardium after systemic infusion of MNBs/Ad_hVEGF_ or MNBs/Ad_GFP_ complexes under magnetic guidance. (A) Immunohistologic analysis for GFP expression in the infarcted myocardium after 48 h and 7 d treatment. Sections were stained with anti-GFP antibody (green) and nuclei were stained with TOPRO3 (red). (B) Sections from the MI-M+MNBs/AdhVEGF group showed several AdhVEGF transduced cells staining positive interspersed in the luminal lining of the vessel. Sections were stained with anti-hVEGF antibody (yellow), anti-CD31 and nuclei were stained with TO-PRO3 (blue). (C) Occasionally Ad_hVEGF_ co-localized with cardiac troponin positive cell. Sections were stained with anti-hVEGF antibody (yellow) and anti troponin T antibody (red). Nuclei were stained with TOPRO3 (red). (MI-M^+^MNBs/Ad_GFP_, n = 4; MI-M^−^MNBs/Ad_GFP_, n = 4).

**Figure 8:**
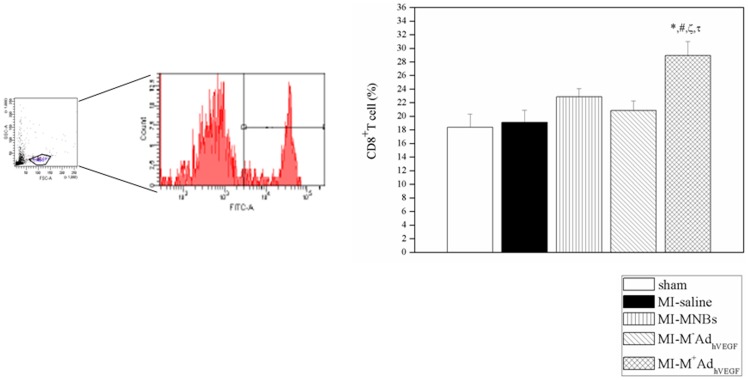
Inflammation response after intravenously injection of MNBs/Ad_hVEGF_ complexes. FACs analyses of mononuclear cells from peripheral blood in different groups. CD8^+^T cells of peripheral blood significantly enhanced in MI-M^+^MNBs/Ad_hVEGF_ compared to sham and other MI treated groups Data ware mean values± SEM. *P<0.05MI-M^+^MNBs/Ad_hVEGF_ versus Sham, #*P*<0.05 versus MI-saline, ζ*P*<0.05versus MI-MNBs and τ*P*<0.05 versus MI-M-AdVEGF. (MI-M^+^ MNBs/Ad_hVEGF_, n = 4; MI-M^−^MNBs/Ad_hVEGF_, n = 4; MI-MNBs, n = 10; MI-Saline, n = 4 and sham-operated, n = 4).

**Figure 9:**
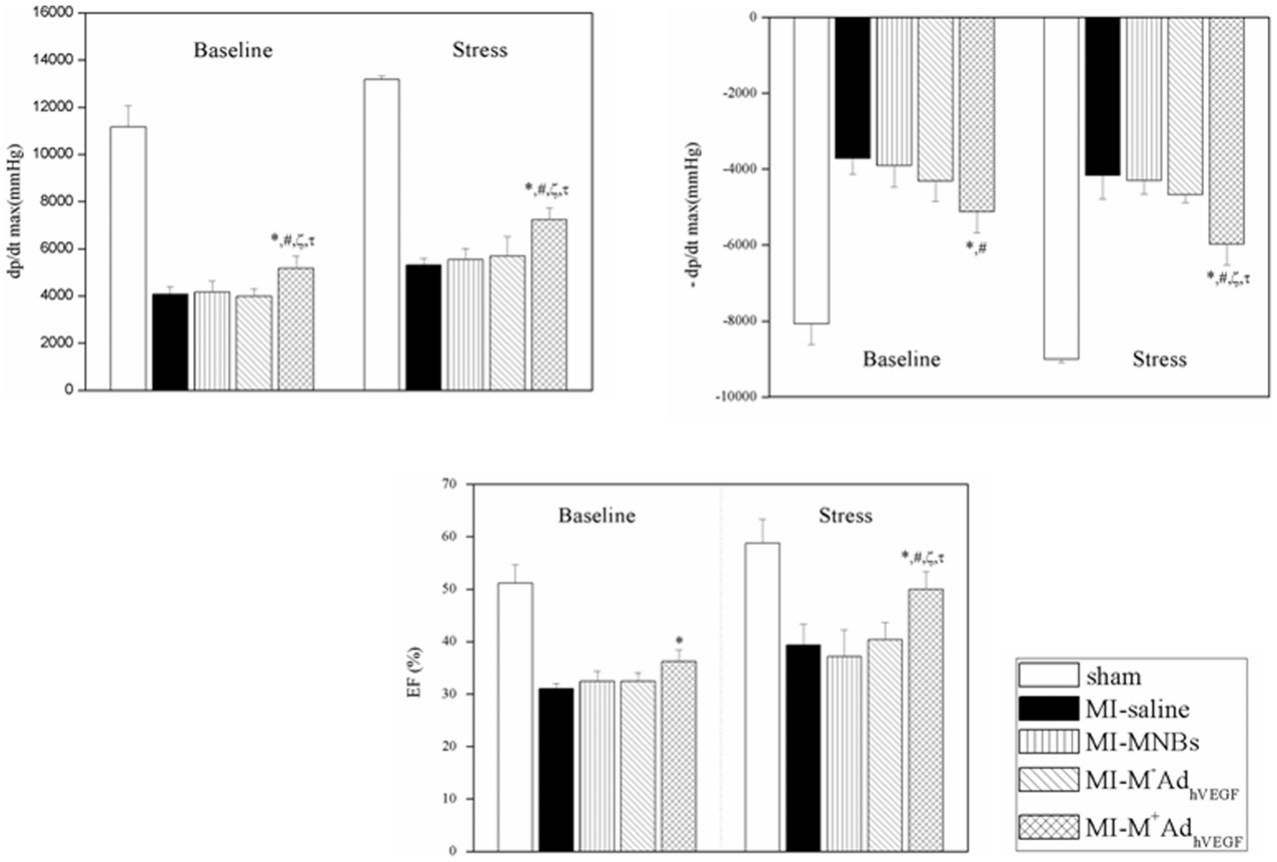
Intravenously MNBs/Ad_hVEGF_ injection restored cardiac function 4 weeks after MI assessed by catherization. Left ventricular function at both baseline and stress conditions revealed the increments of left ventricular dp/dt max and dp/dt min under baseline and dobutamine stress compared with MI-M^−^MNBs/Ad_hVEGF_ group. Meanwhile the ejection fraction (EF) was enhanced under the dobutamine stress compared with the MI-M^−^MNBs/Ad_hVEGF_ group. Data ware mean values± SEM. *P<0.05MI-M^+^MNBs/Ad_hVEGF_ versus Sham, #*P*<0.05 versus MI-saline, ζ*P*<0.05versus MI-MNBs and τ*P*<0.05 versus MI-M-AdVEGF. (MI-M^+^MNBs/Ad_hVEGF_, n = 10; MI-M^−^MNBs/Ad_hVEGF_, n = 10; MI-MNBs, n = 10; MI-Saline, n = 10 and sham-operated, n = 5).

**Table 1:** Hemodynamics of the LV under Baseline condition 4 weeks after MI.

Parameter	Sham (n = 5)	MI-Saline (n = 10)	MI-MNBs (n = 10)	MI-M^−^MNBs/Ad_hVEGF_ (n = 10)	MI-M^+^ MNBs/Ad_hVEGF_ (n = 10)
Pmax (mmHg)	164.39±3.86	92.29±11.86*	90.11±7.31*	93.88±5.24*	120.16±10.64*, #, ζ, τ
dp/dt max (mmHg)	13171.64±147.32	5319.63±270.57*	5547.43±468.45*	5685.61±835.52*	7229.34±501.81*, #, ζ, τ
−dp/dt max (mmHg)	−9008.18±87.54	−4438.80±151.00*	−4300.65±348.98*	−4667.98±218.60*	−5968.96±549.61*, #, ζ, τ
EDV (μl)	198.65±18.81	261.90±9.98*	275.46±10.22*	289.24±11.42*	283.25±11.30*
ESV (μl)	84.51±17.22	155.84±11.74*	162.33±9.91*	172.88±13.50*	145.33±14.26*
SV (μl)	113.66±4.26	105.41±10.65	102.13±12.21	115.42±14.04	138.25±7.3#, ζ
EF (%)	59.78±4.53	39.32±4.00*	37.12±5.11*	40.40±3.29*	49.98±3.34*, #, ζ, τ
HR	439.39±0.77	383.51±3.90	381.22±4.71	384.90±7.37	404.70±9.66

Hemodynamics of the LV under Stress condition 4 weeks after MI.

Values are represented as Mean ± SEM, Pmax means maximum pressure; dp/dt indicates peak rate of maximum pressure rise (dp/dt max) and decline (−dp/dt max); EDV, enddiastolic volume; ESV, endsystolic volume; SV, stroke volume; EF, ejection fraction; and HR, heart rate.

MI-Magnet^+^MNBs/Ad group: a rat myocardial infarction model, a 6×2×2 mm cylindrical NdFeB magnet (Br: ≈1000 mTesla) (IBS, Germany) was placed in the chest of the rat closely adjacent to the infarcted area of the heart and intravenously injected MNBs/Ad complexes.

MI-Magnet^−^MNBs/Ad group: a rat myocardial infarction model, a similar size ceramic bar was placed in the chest of the rat closely adjacent to the infarcted area of the heart and intravenously injected MNBs/Ad complexes.

MI-MNBs group: a rat myocardial infarction model, a 6×2×2 mm cylindrical NdFeB magnet (Br: ≈1000 mTesla) (IBS, Germany) was placed in the chest of the rat closely adjacent to the infarcted area of the heart and intravenously injected MNBs.

Saline group: a rat myocardial infarction model and intravenously injected 0.9% NaCl.

**Figure 10:**
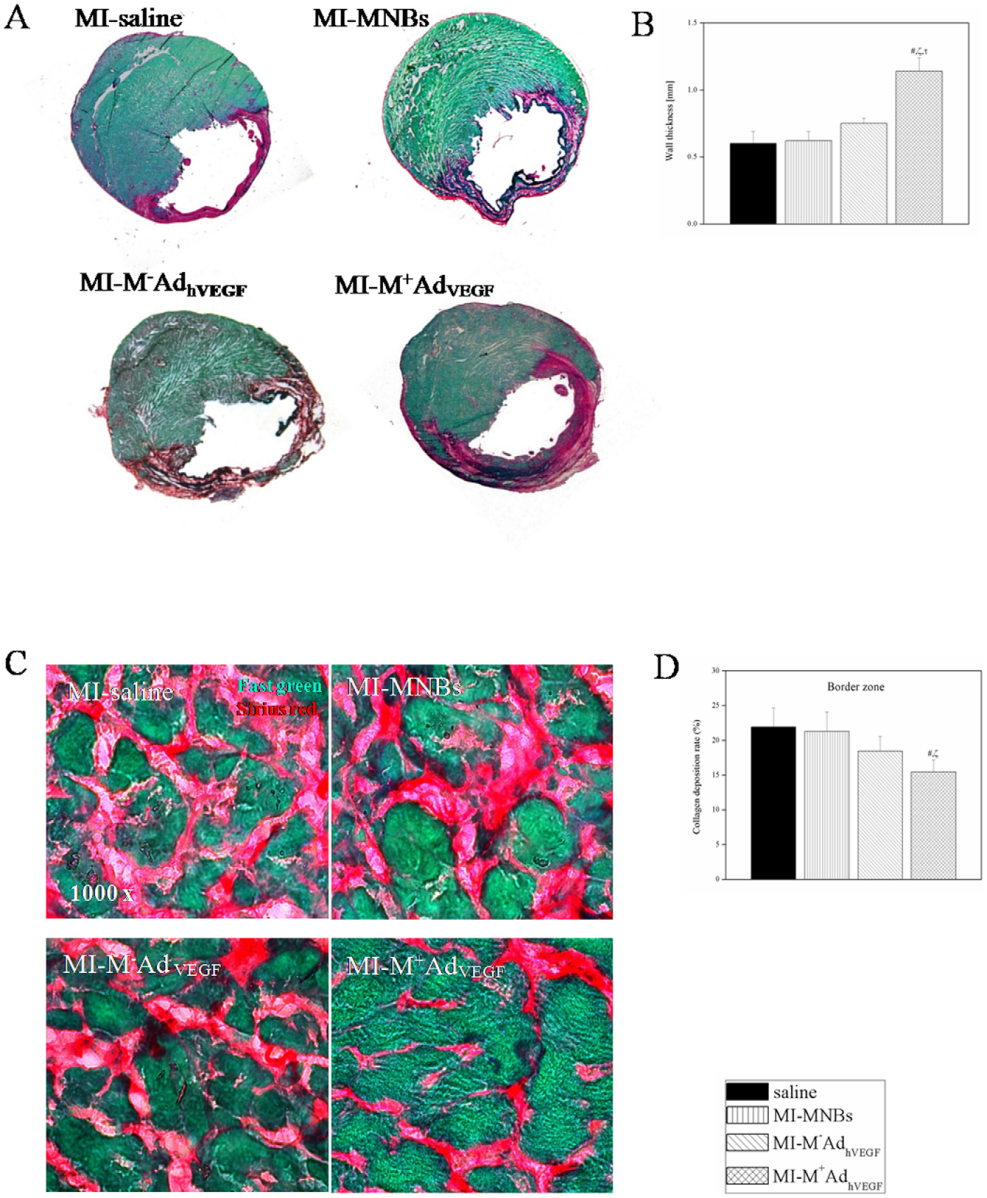
Effects of intravenously MNBs/Ad_hVEGF_ injection on cardiac remodeling 4 weeks after MI. (A) Representative heart cross sections stained with Sirius Red (red, fibrosis) and Fast Green FCF (green, myocyte) from rats. (B) Infarct wall thickness was significantly increased in MI-M^+^MNBs/Ad_hVEGF_ group compared with other MI treated groups. (C) Representative Sirius Red (red, fibrosis) and Fast Green FCF (green, myocyte) staining at border zone. (D) Ratio of collagen deposition is significantly declined in MI-M^+^MNBs/Ad_hVEGF_ compared with other MI treated groups. Data ware mean values± SEM. *P<0.05 MI-M^+^MNBs/Ad_hVEGF_ versus Sham, #*P*<0.05 versus MI-saline, ζ*P*<0.05 versus MI-MNBs and τ*P*<0.05 versus MI-M-AdVEGF. (MI-M^+^MNBs/Ad_hVEGF_, n = 10; MI-M^−^MNBs/Ad_hVEGF_, n = 10; MI-MNBs, n = 10; MI-Saline, n = 10 and sham-operated, n = 5).

**Figure 11:**
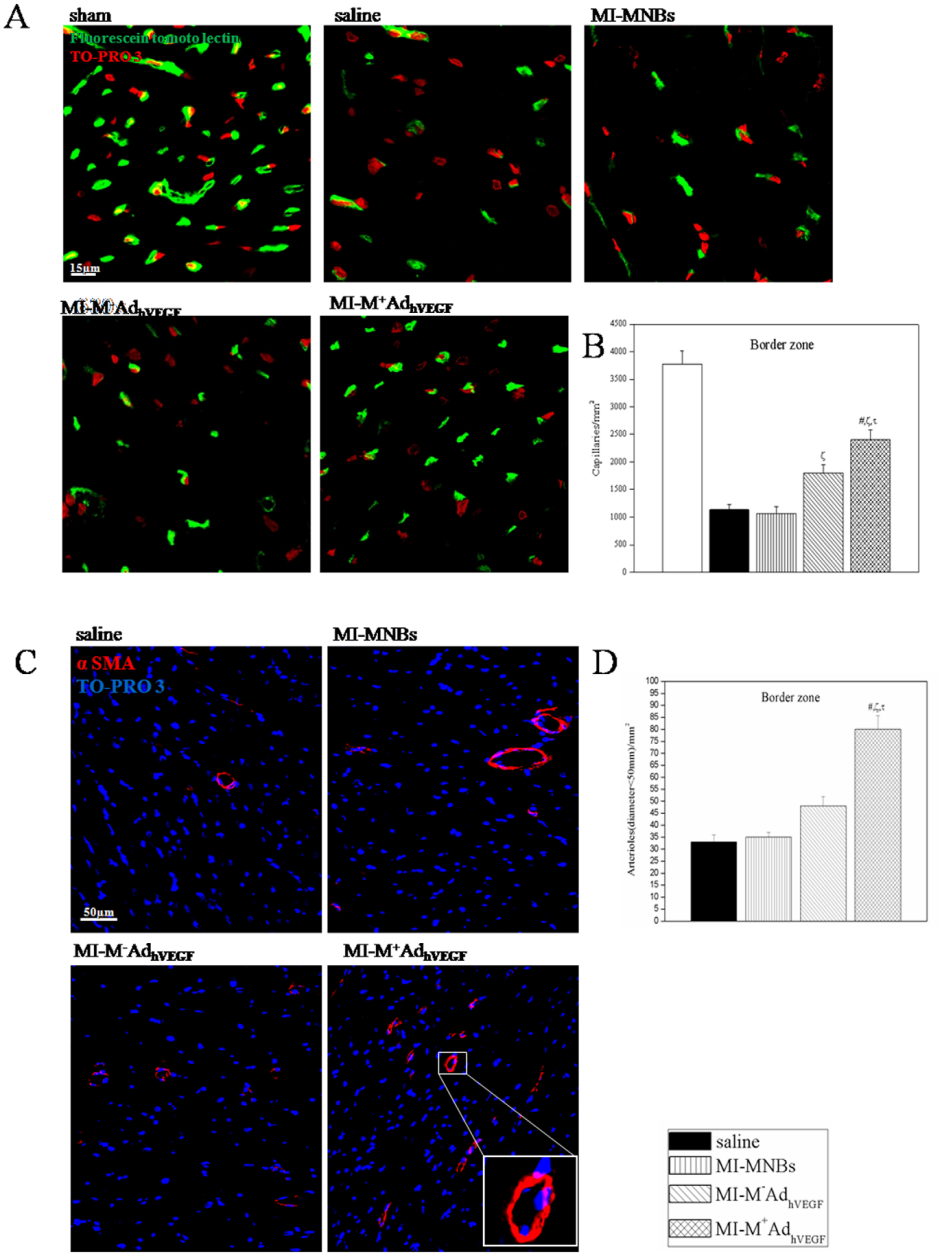
Intravenously MNBs/Ad_hVEGF_ injection induced neovascularization and enhancement of arterioles density 4 weeks after MI. (A) Representative fluorescein tomoto lectin perfusion staining at border area of infarcted hearts. (B) Capillary density in border zone of the left ventricular is significantly higher in MI-M^+^MNBs/Ad_hVEGF_ compared to other MI treated groups. (C) Representative α smooth muscle actin (α SMA) staining at border area of infarcted hearts. (D) Arteriole density at border zone of the left ventricular was significantly higher in MI-M^+^MNBs/Ad_hVEGF_ group compared to other MI treated groups. Data ware mean values± SEM. *P<0.05 MI-M^+^MNBs/Ad_hVEGF_ versus Sham, #*P*<0.05 versus MI-saline, ζ*P*<0.05versus MI-MNBs and τ*P*<0.05 versus MI-M-AdVEGF. (MI-M^+^MNBs/Ad_hVEGF_, n = 10; MI-M^−^MNBs/Ad_hVEGF_, n = 10; MI-MNBs, n = 10; MI-Saline, n = 10 and sham-operated, n = 5).

### FACs analysis for inflammation response

Analysis of peripheral blood were performed at 7 d (n = 4, for each group) after MI. To examine the effects of Ad treatment on inflammation, mononuclear cell fraction of peripheral blood was separated for flow cytometry analysis. Then cells were incubated with anti-rat CD8^+^T monoclonal antibody (Santa Cruz, USA). Subsequently, donkey anti rat Alexa-Fluor 488 conjugated secondary antibody (Molecular probes^TM^) was applied to samples. The number of CD8^+^T cells in the mononucelar cell fraction of peripheral blood was examined by FACs (Calibur, Becton Dickinson, San Jose, USA).

### Quantitative Real Time-PCR and immunostaining analysis for Ad expression and distribution

To investigate the Ad expression and distribution in vivo, mRNA levels of Ad_hVEGF_ were evaluated in different organs. Seven days after Ad injection, hearts, livers, spleens, lungs and kidneys from 4 rats in each group were collected and snap-frozen in liquid nitrogen. Total RNA was isolated following the instructions of the TRIZOL® Reagent (Invitrogen) including DNase treatment. Human VEGF (TaqMan® Gene Expression Assay, Applied Biosystems, USA) primer was used. Amplification and detection were performed with the StepOnePlus™ Real-Time PCR System (Applied Biosystems, USA) in TaqMan Universal Master Mix (Applied Biosystems, USA) according to the instructions of the manufacturer (Applied Biosystems, USA) and repeated at least three times using the following program: 1 cycle of 50°C for 2 min, 1 cycle of 95°C for 10 min, and 40 cycles of 95°C for 15 s and 60°C for 1 min. cDNA extracts were tested in at least triplicate and negative controls were included in each assay. Cycle thresholds (C_T_) for single reaction were determined with StepOne™ Software 2.0 (Applied Biosystems) and the target genes were normalized against GAPDH (formula: ΔC_T_ = C_T target_−C_T GAPDH_ ). Resulting ΔC_T_ of triplicates was averaged and ΔΔC_T_ were obtained using Sham group as calibrator sample (formula: ΔΔC_T_ = ΔC_T sample_−ΔC_T calibrator sample_). In the current study, the 2^−ΔΔC^ method was employed to present the changes in gene expression.

For immunohistochemistry detection of hVEGF and GFP expression, frozen transverse tissue sections (8 µm) of hearts from MI-Magnet^+^MNBs/Ad_hVEGF_; MI-Magnet^−^MNBs/Ad_hVEGF_; MI-Magnet^+^MNBs/Ad_GFP_ and MI-Magnet^−^MNBs/Ad_GFP_ (n = 4 for each group) were incubated with rabbit anti-hVEGF antibody (R&D, USA) or goat anti-GFP conjuganted FITC antibody (Abcam, UK). Subsequently, the sections of Ad_hVEGF_ groups were incubated with donkey anti rabbit Alexa-Fluor 405 (Molecular probes^TM^) conjugated secondary antibody. Nuclei were counterstained with TO-PRO3 (Molecular probes^TM^). Labelled sections were observed using a Leica SP2 Confocal Microscope (Leica, Germany).

Moreover, in order to identify cell types infected by Ad_hVEGF_ in the infarcted heart, immunohistochemistry staining was performed. Polyclonal goat anti-CD31 (Santa Cruz, USA) primary antibody was initially applied to the section followed by anti goat Alexa-Fluor 488 (Molecular probes^TM^) secondary antibody incubation. Subsequently, human VEGF was stained following the protocol previously described and a goat polyclonal anti-Troponin T primary antibody (Santa Cruz, USA) was applied to the sections. A donkey anti-goat Alexa-Fluor 568 (Molecular probes^TM^) was utilized during secondary antibody reaction. Counterstaining was achieved by TO-PRO-3 (Molecular probes^TM^) nuclear staining. The samples were analyzed using a LSM 780 confocal microscopy (Carl Zeiss, Jena, Germany).

### Left ventricular catheterization for heart function evaluation

4 weeks after surgery, rats underwent pressure-volume (P/V) loop measurements according to the protocol of CardioDynamics BV (CD Leycom, Zoetermeer, Netherlands). Data were collected with the Millar Pressure-Volume System (Ultra-Miniature Pressure-Volume Catheter (model SPR-1030), Millar Pressure Conductance Unit (model MPCU-200) and Millar PowerLab data-acquisition hardware; emka Technologies, Paris, France). Calibration of pressure and volume was performed by equating the minimal and maximal conductances with minimal (0 mmHg) and maximal (100 mmHg) pressures as well as minimal and maximal blood volumes received from venous circulation. After inserting the catheter into the carotid artery, retrograde access to the left ventricle (LV) was achieved. P/V loops were recorded under normal conditions (baseline) followed by stress conditions mediated by intravenous dobutamine administration (10 µg/kg/min, Sigma-Aldrich, Deisenhofen, Germany). Volume signal was corrected by measurement of wall conductance (parallel volume) via hypertonic saline (5%) injection. Data were analyzed with IOX Version 1.8.3.20 software (emka Technologies). As histological evaluation on hearts in different cardiac phases can lead to under- or overestimation of the analysis such as capillary density, infarct size, fibrosis etc, after P/V loop measurements, rats were euthanized by 5% KCl perfusion. The overdose of potassium chloride may cause cardiac conduction blocks and stop the heartbeat at the diastolic phase so that the histological analysis was performed in the diastolic phase of the heart.

### Determination of functional perfusion

Hearts were removed and after cannulation of the aorta they were perfused retrogradely at constant pressure in the Langendorff mode with 7.5 mg/ml of Fluorescein Lycopersicon esculentum (tomato) lectin (LINARIS; Wertheim-Bettingen, Germany) suspended in 0.2-mm-filtered Krebs-Henseleit (KH) buffer (117 mM NaCl, 24 mM NaHCO3, 11.5 mM D-[t]-glucose, 3.3 mM KCl, 1.25 mM CaCl2, 1.2 mM MgSO4 and 1.2 mM KH2PO4) equilibrated with 95% O2 and 5% CO2 followed by 20 ml kH buffer alone. Tomato lectin binds to the surface N-acetylglucosamine oligomers of endothelial cells lining perfused vessels, thereby delineating perfused vasculature [19]. Direct contact of tomato lectin with endothelial cells is required for labeling. Therefore, vessels that are not perfused will not be labeled with tomato lectin. Finally hearts were embedded in O.C.T TM Compound (Tissue-Tek®; Zoeterwoude, Niederlande) and snapfrozen in liquid nitrogen. To prepare the myocardial tissue for histological and biomolecular investigations the infarct area has been divided into 4 horizontal levels from top to bottom within each given amount of 8 mm sections was cut.

### Infarction wall thickness and fibrosis analysis

Heart sections of 4 horizontal infarct levels (8 µm) were stained with Fast Green FCF (Sigma-Aldrich) and Sirius Red (Division Chroma, Germany). The infarcted wall thickness was analyzed using computerized planimetry (Axio Vision LE Rel. 4.5 software; Zeiss, Germany). To evaluate fibrosis the Sirius red positive regions of collagen deposition in the border zone near infarcted area were examined in 10 randomly chosen fields per section (one section per level; 1000×) using computerized planimetry. Collagen density was expressed as the ratio of collagen deposition to myocardial tissue in percentage.

### Determination of capillary density and arteriole density

Tomato lectin perfusion of the hearts as described was used for analysis of capillary density. Heart sections of two contiguous levels of the heart which represent the major infarct region were stained with TO-PRO3 (Molecular probes^TM^). The sections were analyzed within the border zone of the heart. Capillary density was assessed by counting the number of capillaries in 5 border zone randomly chosen fields (630×). For immunohistochemistry detection of arteriole density, heart sections of two contiguous levels of the heart which represent the major infarct ratio were immunohistochemistry staining with polyclonal rabbit anti-α smooth muscle actin (αSMA) (Abcam, UK) primary antibody followed by goat anti rabbit Alexa-Fluor 568 (Molecular probes^TM^) conjugated secondary antibody and counterstained with TO-PRO3 (Molecular probes^TM^). The sections were analyzed with in the border zone of the hearts. Arteriole density was assessed by counting the number of arterioles in 5 border zone randomly-chosen fields (400×). Results were expressed as capillaries/Aeterioles per mm^2^.

Tomato lectin perfusion of the hearts as described was used for analysis of capillary density. Heart sections of two contiguous levels of the heart which represent the major infarct region were stained with TO-PRO3 (Iinvitrogen, USA). The sections were analyzed within the border zone of the heart. Capillary density was assessed by counting the number of capillaries in 5 border zone randomly chosen fields (630×). For immunohistological detection of arteriole density, heart sections of two contiguous levels of the heart which represent the major infarct ratio were immunostained with polyclonal rabbit anti-αSMA (Abcam, UK) primary antibody followed by anti rabbit Alexa-Fluor 568 (Invitrogen, USA) conjugated secondary antibody and counterstained with TO-PRO3 (Invitrogen, USA). The sections were analyzed with in the border zone of the hearts. Arteriole density was assessed by counting the number of arterioles in 5 border zone randomly-chosen fields (400×). Results were expressed as capillaries/Aeterioles per mm^2^.

### Statistical analyses

All data are expressed as mean values ± SEM or values ± SD. One way analysis of variance was employed for comparing differences between groups. Least significant difference (equal variances) and Dunnett's T3 (non-equal variances) post hoc tests were used for testing the differences between groups. All tests were two-tailed, and significance was accepted at *P*<0.05.

## Results

### MNBs/Ad complexes characterization

After the formulation of MNBs/Ad complexes, the average diameter of these particles was assessed by ZetaPALS analyzer. The mean diameter of naked Ad (100–200 nm) detected was larger than the actual size of adenovirus (∼90 nm), mainly because of fixed aqueous layer formed by hydration of the adenoviral particle in the solution. The size of MNBs/Ad (300–600 nm) was larger than the naked Ad, which due to MNBs forming a shell around the viral particle. Putting 8 µl Ad with concentration 8×10^7^ pfu/cm^2^ in different tubes, we added different doses of MNBs to prepare different MNB/Ad ratios of complexes. Figure 2 indicated that with increasing the dose of MNBs, the group of MNBs/Ad complexes was increased whereas the naked Ad group was reduced. After that, the MNBs/Ad complexes group became close to constant 100% from the MNBs dose at 16 µl. This result proved that all Ad have been bound with MNBs at MNBs dose of 16 µl, Ad concentration at 8×10^7^ pfu/cm^2^, and MNBs/Ad ratio is 2.

### Transduction efficiency *in vitro*

The transduction efficiency of MNBs/Ad_luc_ complexes was assessed using luciferase as the report gene (Figure 3). The transduction efficiency mediated by different quantities of MNBs and a constant amount of Ad_luc_ (adenoviral vector concentration: 1×10^10^ pfu/ml) was compared with Ad_luc_ alone (MNBs dose = 0 µl) in HUVECs subjected to 30 min incubation under a magnet field. It was found that conjugation of MNBs to Ad_luc_ complexes significantly increased the transduction efficiency and resulted in the highest enhancement in transduction efficiency at 16 µl MNBs, 8×10^7^ pfu/cm^2^ Ad and a MNBs/Ad ratio of 2. The peak transduction efficiency was 50 fold higher than that offered by Ad_luc_ alone. The peak transduction efficiency was 50 folds higher than that offered by Ad_luc_ alone.

### *In vitro* cytotoxicity of MNBs/Ad complexes

The *in vitro* cytotoxicity of MNBs/Ad_luc_ was evaluated in HUVECs using MTT [3- (4,5-dimethylthiazol-2-yl)-2,5-diphenyl tetrazolium bromide] cell proliferation assay. Relative viabilities (MNBs/Ad_luc_ relative to Ad_luc_ alone) indicated that the cytotoxicity increased by enhancing MNBs dose or Ad amount (Figure 4). However, the optimal parameters of MNBs/Ad (16 µl MNBs, 8×10^7^ pfu/cm^2^ Ad and a MNBs/Ad ratio of 2) still kept a comparatively higher cell viability above 85% (Figure 3), suggesting the optimized parameter of MNBs/Ad which owned highest transduction and relatively low cytotoxicity adapted to gene transduction *in vitro* or *in vivo*.

### Magnetic field guidance *in vitro*

X-gal staining showed that reporter gene expression formed a well-defined three spots pattern and was confined to the areas where the magnets had been attached prior to the administration of MNBs/Ad_luc_ complexes (Figure 5A). Microscopic evidences for the LacZ reporter gene expression in the confined area adjacent to the magnet and in the area in the absence of the magnetic field are shown in Figures 5B and 5C, respectively. Meanwhile a clear border zone were shown between magnetic and non-magnetic area (Figure 5D). The transduction efficiency in the area adjacent to the magnet was more than 80% and in an area in the absence of the magnetic field was less than 4%. In the context of *in vitro* application, this phenomenon offered a potential use of a magnetic field to control the localization of gene expression in complex biological systems.

### Distribution and expression of Ad in infarcted rat hearts

We examined the distribution and expression of systemic delivery of MNBs/Ad_hVEGF_ 7 d after intravenous injection. Here, a significantly higher hVEGF expression in hearts was found in the MI-M^+^MNB_S_/Ad_hVEGF_ group compared to the MI-M^−^MNB_S_/Ad_hVEGF_ group (Figure 6). We also checked the hVEGF expression in liver, spleen, lung and kidney. However, the hVEGF expression in these organs was significantly lower in the MI-M^+^MNB_S_/Ad_hVEGF_ group compared to the MI-M^−^MNB_S_/Ad_hVEGF_ group (Figure 6).

Immunohistochemistry staining with GFP revealed that most of the Ad_GFP_ was expressed in the border zone of the ischemic myocardium (Figure 7A) both 48 h and 7 d after intravenous injection. This phenomenon suggested that magnet may successfully attract MNBs/Ad to magnetic field area/border zone of infarcted myocardium. Furthermore, double staining with anti-hVEGF and anti-CD31 (Figure 7B) showed that hVEGF was expressed in the luminal side of the vessel which indicated that hVEGF may play a role in repair of endothelial cells lost by ischemic injury. In addition we observed that a small number of cardiac Troponin T(cTnT) positive cells can express hVEGF (Figure 7C).

### Inflammation analysis

Systemic delivery of MNBs/Ad enhanced CD8^+^T cell mobilization to the peripheral blood at 7 d. There was a moderate increase of the mean percentages of CD8^+^T cells in the mononuclear cells of peripheral blood in MI-M^+^ MNBs/Ad_hVEGF_ comparison to sham and other MI treated groups (Figure 8), indicating more complexes of MNB/adenovirus were attracted to the heart by the magnetic guiding. The accumulated virus in the heart would induce inflammation response which leads to the enhancement of CD8+ T cells in the heart, spleen and peripheral blood [27].

### Cardiac function

Hemodynamic measurement of the cardiac performance (Figure 9, Table 1) demonstrated an improvement of functional parameters in case of MI-M^+^MNBs/Ad_hVEGF_ treatment both under baseline conditions and after stress induction. The values of the maximum and minimum first derivatives of LV pressure (d*P*/d*t*max and d*P*/d*t*min) were significantly lower in the infarcted animals than in the sham animals (Table 1). However, d*P*/d*t* max was markedly enhanced in the MI-M^+^MNBs/Ad_hVEGF_ group compared to the MI-M^−^MNBs/Ad_hVEGF_ group and other MI treated groups both under baseline and stress condition (Figure 9). Furthermore, MI-M^+^MNBs/Ad_hVEGF_ treatment enhanced systolic and diastolic properties of the infarcted LV, and it produced an obvious increased LV ejection fraction compared to MI-M^−^MNBs/Ad_hVEGF_ and other MI treated groups under stress condition (Figure 9). Significant values have also been found regarding the maximum pressure (Pmax) (Table 1).

### Infarction wall thickness and fibrosis

Left anterior descending (LAD) ligation consistently resulted in transmural myocardial infarction, exhibiting typical histologic changes including thinning of the left ventricular free wall (Fast green), extensive collagen deposition (Sirius red), progressive ventricular chamber dialation, hypertrophy, fibrosis and prolonged cardiomycyte apoptosis. Fibrosis resulted in extensive collagen deposition (Sirius red) and increased distance between myocytes (Fast green) 4 weeks after myocardial infarction (Figure 10).

Representative heart sections of 4 weeks after myocardial infarction in different groups are shown in Figure 10. The mean infarct wall thickness was significantly increased in the MI-M^+^MNBs/Ad_hVEGF_ group compared to the MI-M^−^MNBs/Ad_hVEGF_ and other MI treated groups, (Figure 10A and B). The histomorphological appearance of the border zone indicated a higher portion of collagen deposition in the MI treated groups (Figure 10C). However, there was marked declined collagen deposition in the MI-M^+^MNBs/Ad_hVEGF_ group (Figure 10D).

### Capillary and arteriole density

Capillary and arteriole density was analyzed by immune staining (Figure 11A & C) 4 weeks after MI. As expected, there was a marked decrease in capillary and arteriole density in the border zone in the infarcted animals. However, compared with other MI treated groups the capillary and arteriole density was significantly higher in infarct border zone in the MI-M^+^MNBs/Ad_hVEGF_ group (Figure 11B & D). Capillary and arteriole density enhancement may support cardiomyocyte survival by supplying necessary oxygen and nutrition.

## Discussion

In this study we developed stable magnetic nanobead/Ad complexes and administered the complexes via intravenous injection for targeted delivery of therapeutic genes to ischaemically damaged hearts in a rat acute myocardial infarction model for AMI treatment under the guidance of the magnetic field.

We utilized a stable and tight attachment that coupled Ad to MNBs by employing the high binding affinity of the biotin–streptavidin interaction [28]–[30]. *In vitro* experiment, we optimized the conjugation ratio of Ad_luc_ to MNBs. At the optimal conjugation ratio, the peak transduction efficiency was 50 fold higher than that offered by Ad_luc_ alone. Meanwhile the MNBs/Ad_lacZ_ complexes could be directed to specific locations defined by external magnets. *In vivo,* the epicardial magnet effectively attracted MNBs/Ad_hVEGF_ complexes and resulted in strong therapeutic gene expression in the ischemic zone of the heart. And then it promoted angiogenesis and improved heart function. It follows, therefore, the systemic administration of MNBs/Ad by external magnetic control may be a useful, non-invasive, gene-based therapy to enhance post–infarction myocardial repair.

A series of research proved that *in vitro*, MNBs based infection increased sedimentation of the complex and the speed and efficiency of transfection. And *in vivo,* the therapeutic gene may be expressed in target site/organ in the body and enhance the transduction efficiency by magnetic field guiding. Pandori M and colleagues [31] utilized the streptavidin and its ligand biotin to associated Ad to silica beads result in higher infection efficiency for a variety of cell lines and a highly localizable by utilization of magnetic force. Moreover Hofmann A *et al* [14] used charge to couple magnetic nanoparticles (MNPs) to lentiviral vectors (LVs) and applied MNPs to combine cell transduction and positioning in the vascular system. In addition, *in vivo*, MNPs/LVs biodistribution is significantly changed by external magnetic fields intervention in mice model. In our system, we utilized high affinity and effective binding properties of biotin and streptavidin to form the extremely stable magnetofection complexes. *In vitro*, compared with Ad_luc_ alone, MNBs/Ad_luc_ complexes had the 50 fold higher transduction efficiency under the magnetic field control. *In vivo,* in rat MI model, 7 days after intravenous injection MNBs/Ad_hVEGF_ or MNBs/Ad_GFP_ complexes, the higher level of hVEGF or GFP gene expression were detected by RT-PCR and immunostaining in the ischemic myocardium in magnet positive group compared to the magnet negative groups. Our results were consistent with our previous study [15] which demonstrated that application of an external magnet were able to alter the biodistribution of systemically administered MNBs-coupled non-viral vectors, and to prove MNBs assisted transfection resulted in efficient site-directed targeting of the heart endothelium.

It may be a matter for concern that combined MNBs to Ad to transduce cells *in vitro* and *in vivo* would influence their phenotype and viability. In our experiment MNBs/Ad transduced HUVECs had typical endothelial cells morphology as non-transduced HUVECs and no significant change in the viability of HUVECs was observed at the optimal conjugated ratio of Ad to MNBs. It was consistent with Myokhayly O and colleagues [32] result which showed that there was no side effect on MNBs induced cell magnetofection.

A lot of studies have investigated gene therapy for post-myocardial infarction. VEGF plays a vital role for angiogenesis and neovascularization in physiological and pathological aspects. After MI, the ischemic heart muscle can express and secrete VEGF for neovascularization [33] and VEGF has been comprehensive application for repair ischemic myocardium because it may benefit remodeling and function [34], [35]. Hence, delivery of viral vectors encoding vascular endothelial growth factor gene has showed great potential in cardiac regeneration after MI. In our study we demonstrated systematic administration Ad_hVEGF_ improved right ventricular performance, 7 days after infarction, coincident with the detection of hVEGF in heart tissue, MI-M^+^MNBs/Ad_hVEGF_ transduced rats group showed hVEGF lining expression in the lumen of vessel. This result indicated that hVEGF could be successfully expressed in endothelial cells and it is well known that VEGF strongly induces the activity of extracellular signal-regulate kinases(ERKs) and activation of this pathway plays an important role in the stimulation of endothelial cell proliferation and further for neovascularization [36]. In addition significant increase in the angiogenesis was occurred in the border zone of ischemic heart. Similar findings were reported by Crottogini A and Siddiqui AJ groups [7], [37] who showed that hVEGF induced neovascularization in rats and pigs. This was consistent with the finding from Guerrero M group [38], in which intracardiac injection hVEGF induced gene expression on cardiac tissue and promoted neovascularization in the infarct area. The enhancement in capillary density of the infarct zone may partially account for the improved LV function in gene-treated animals.

Although the technique described in the present study was a promising step towards magnetically controlled gene delivery for MI, many questions and problems remain to be addressed before it becomes a clinically useful tool. Firstly during this study, 7 days after intravenous injection we found a moderate enhancement of CD8^+^ T cells mobilized to peripheral blood compared with MI-saline and MI-Magnet^−^MNBs/Ad_hVEGF_ groups. CD8^+^ T cells play an important role in host defense against viral infections. Also CD8^+^ T cells could produce interferon-*γ* (IFN-*γ*), tumor necrosis factor-*α* (TNF-*α*) and interleukin-2 (IL-2), which are able to induce apoptosis cell death [39].

Another obviously disadvantage was Ad mediated gene expression can not be expressed for a long time because the adenoviral genome did not insert host chromosome during the replication. Therefore long-term expression and low immunogenecity need to be achieved by other gene carriers. Adeno-associated virus (AAV) is small nonenveloped, single-stranded DNA viruses that can potentially infect both dividing and non-dividing cells and may integrate in the genome of the host cell, making it possible to achieve a long-term transgene expression. Especially AAV serotype 9 (AAV9) has been shown to have a preferable myocardium transduction after systemic administration, making it ideal for non-invasive therapeutic gene transfer of the heart. Previous studies have proved that AAV9 has the potential function of over expression therapeutic gene in the heart for cardiac regeneration [40], [41].

## Conclusions

In summary, we showed that systemic delivery of MNBs/Ad_hVEGF_ complexes under external magnetic guidance could increase the VEGF expression in the infarcted myocardium, leading to neovascularization and improved post-infarction recovery of LV function. Intravenous delivery of MNBs-based gene therapy may be a useful strategy for ischemic heart disease treatment in patients and have a great application meaning in clinical research.
